# Pedicled Radial Forearm “Free” Flap for Intraoral Reconstruction Based on an Unexpectedly High Origin of the Radial Artery—Case Report

**DOI:** 10.3390/clinpract15010006

**Published:** 2024-12-30

**Authors:** Marino Lupi-Ferandin, Dinko Martinovic, Ante Pojatina, Ante Mihovilovic, Ema Puizina, Sasa Ercegovic, Ivana Stula, Josko Bozic, Slaven Lupi-Ferandin

**Affiliations:** 1 Study of Dental Medicine, University of Split School of Medicine, 21000 Split, Croatia; 2 Department of Maxillofacial Surgery, University Hospital of Split, 21000 Split, Croatiaslupif@kbsplit.hr (S.L.-F.); 3 Department of Radiology, University Hospital of Split, 21000 Split, Croatia; 4 Department of Pathophysiology, University of Split School of Medicine, 21000 Split, Croatia

**Keywords:** squamous cell carcinoma, radial forearm free flap, vascular aberration

## Abstract

**Background:** Radial forearm free flap (RFFF) is considered one of the workhorses in modern head and neck reconstruction surgery due to its technical simplicity, versatility and less time-consuming harvest. **Methods:** In this report, we present the case of a 56-year-old woman with sublingual squamous cell carcinoma (SCC) who underwent surgical resection and reconstruction of the defect with a RFFF. **Results:** The preoperative Allen test showed normal blood flow, and the ultrasound did not recognize any blood vessel abnormalities in the left arm. However, during the RFFF harvest, when the dissection of the pedicle came to the cubital fossa, there was no brachial artery bifurcation. While trying to find the bifurcation, the dissection almost came to the axillary region. Hence, the RFFF was converted to a pedicle flap and was pulled through to the intraoral defect where it was used for reconstruction. **Conclusions:** Hence, during the preoperative radiological ultrasound, besides the usual characteristics such as the radial artery diameter, flow and possible obstructions, it is also important to explore if there are any other anatomical abnormalities that could influence the operation.

## 1. Introduction

Today, microsurgical free flaps are considered the “golden standard” for head and neck reconstruction surgeries regardless of the tissue defect etiology, whether it be excision of malignancies or traumatic wounding [1]. Furthermore, the advancement of microsurgery during the last several decades has formed the foundation for modern head and neck reconstructive surgery. One of the most popular free flaps, not only for head and neck reconstruction but other regions as well, is the radial forearm free flap (RFFF) [2]. The RFFF was first described in 1981 by Yang et al., and the initial indication was a burn scar contracture relief [3]. It was also named the Chinese flap after the first authors. Nowadays, it is still one of the most commonly used free flaps in head and neck reconstruction due to its advantages such as the long vessels, pliable skin, relatively sufficient diameter of the artery and easy harvest [1]. These features make it particularly suitable for reconstructing complex defects in areas such as the oral cavity, pharynx and facial regions. The flap’s excellent vascularity supports high survival rates, often exceeding 98% in highly educated centers, and its adaptability allows for use in various defect sizes [4,5]. However, on the other hand, the RFFF is associated with significant donor site morbidity [5,6]. Common complications include poor aesthetic outcomes, such as scarring and tendon exposure, as well as functional deficits like reduced grip strength or range of motion [6]. In rare cases, ischemia or fractures of the radius may occur if bone is included in the flap [1]. The need for skin grafting at the donor site further increases the risk of infection or delayed healing. While preoperative assessments like the Allen test mitigate vascular risks, sacrificing the radial artery remains a drawback.

The main blood supply for the RFFF is conducted through the radial artery, which is a branch of the brachial artery, the other branch being the ulnar artery. The brachial artery usually bifurcates about 1 cm distal to the antecubital fossa and the radial artery runs from this bifurcation deep to the brachioradialis muscle proximally. The remainder of the artery is superficial, covered by the fascial coverings and skin. The vascular supply of the forearm skin runs through the septocutaneous perforators. The radial artery is accompanied by two venae comitantes that represent the main venous drainage of the RFFF. The superficial venous system through the cephalic vein also represents an important flap venous drainage.

Prior to the free flap era, pedicled regional flaps were the workhorses in head and neck reconstruction and they were mostly used for oncological indications. Some of them are the pectoralis major musculocutaneous flap, latissimus dorsi musculocutaneous flap, deltopectoral flap and forehead flap. While it is established that these flaps are inferior compared to the free flaps, on the other hand, they can still be useful due to several reasons: lack of microsurgical expertise; free flap failures; lack of adequate vessels for anastomosis as a consequence of radiotherapy; free flap main vessels obstruction/stenosis; patient age and comorbidities [7,8].

In this report, we present a case of sublingual squamous cell carcinoma (SCC) which underwent surgical resection and reconstruction of the defect with a RFFF. However, there was an anatomical variation in the left hand where the patient did not have a brachial artery bifurcation. Hence, while dissecting proximally to find the bifurcation we almost came to the axillary region and decided to convert the RFFF to a pedicled flap.

## 2. Case Report

A 56-year-old woman was referred to our department due to intraoral pain. She had a history of tobacco and alcohol consumption. Upon examination, an ulceration was observed in the left sublingual area, and it was crossing the medial line. Radiological findings using the MSCT did not show any pathological lymph nodes in the neck. The pathohistological report of the biopsy confirmed SCC and the staging of the cancer was T3N0M0. The oncological multidisciplinary team decided that primary surgical management was the best treatment option. The surgical plan was to perform a bilateral selective supraomohyoid neck dissection and en bloc resect the tumor via the “pull through” approach. The reconstruction was planned with a RFFF. A preoperative ultrasound performed by a radiologist showed a normal anatomy as well as diameter and flow of the forearm arteries. Also, the preoperative Allen test showed regular blood flow and communication between the ulnar and radial vascular systems.

On the day of the operation, after the induction into general anesthesia, nasal intubation and catheterization of the bladder, the operating fields were prepared according to the usual practice. The neck dissection and tumor excision were conducted by one team of surgeons according to the usual practice. The other team conducted the harvest of the RFFF.

The outlines of the RFFF were marked on the left forearm skin, and then dissection started. The flap was raised in a regular manner from the ulnar to the radial side and the cephalic vein was included in the flap as well. The radial artery and accompanying venae commitantes were in their regular positions between the brachioradialis muscle and the flexor carpii radialis muscle. After the flap dissection, a longitudinal incision was made up to the antecubital crease, the skin flaps were raised and the dissection of the flap pedicle was conducted. Furthermore, along with the flap pedicle, the cephalic vein was harvested as one more possibility for venous anastomosis.

The dissection proceeded proximally up to the cubital fossa, where the dissection of the brachial artery bifurcation is performed. However, we came upon an unusual situation. Instead of a normal bifurcation we only noticed the radial artery. Moreover, we regularly perform dissection proximally to the cubital fossa because we seek bigger caliber veins (communications between deep and superficial vein system) that are more convenient for venous anastomosis.

**Figure 1:**
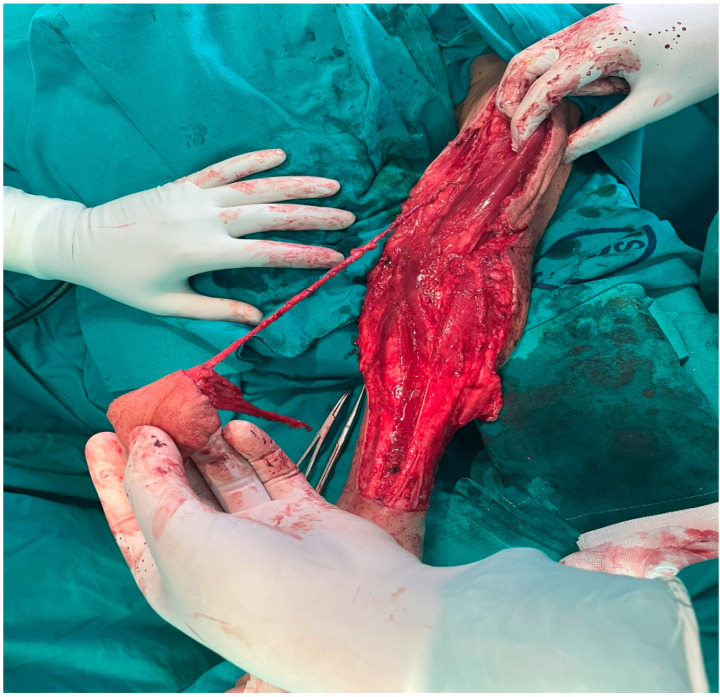
The radial forearm “free” flap with the pedicle up to the axillary region.

In the light of this new situation, we decided to proceed with the dissection proximally to further explore the unusual anatomy and find the bifurcation. The radial artery and the cephalic vein, the pedicle of the flap, were dissected till the axillary region and we did not encounter any branching of the artery (Figure 1). Hence, due to the unusually long pedicle, we decided to move the “RFFF” as a pedicled flap to the defect. We undermined and tunneled the skin and the subcutaneous layer in the supraclavicular and cervical region, and the pedicled “RFFF” was pulled through into the oral cavity to reconstruct the defect. The donor site of the flap was closed in the usual practice with a split-thickness skin graft harvested from the buttock.

**Figure 2:**
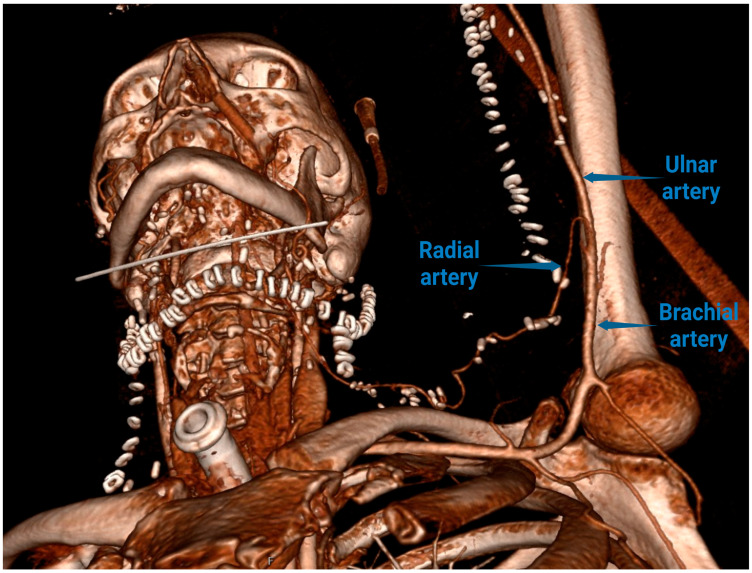
Radial artery branching from the brachial artery almost at the axillary region and following the direction of the radial flap in the oral cavity.

The postoperative recovery was uneventful, and the radial flap survived without any complications. During the postoperative course, we performed MSCT angiography that showed the high origin of the radial artery as a part of the 40 cm long flap pedicle (Figure 2). The patient was discharged on the 23rd postoperative day and underwent adjuvant radiotherapy. Later, she developed osteoradionecrosis of the lower jaw and underwent stem cell therapy at our department. Today, she is alive and entirely functional with the radial flap fully vital.

## 3. Discussion

Pedicled radial forearm flaps are usually used for hand, wrist or elbow reconstructions [9,10]. Furthermore, pedicled radial forearm flaps can be used for distant locations as a two-act surgery where, in the second surgery, the division of the pedicle is conducted [11]. However, with extensive research through the literature, we were not able to find a similar case report where a RFFF was, due to the radial artery’s high origin, used as a one-act pedicled flap for head and neck reconstruction. Nevertheless, it was described that in some situations, like that of previously operated or irradiated necks, when there are no appropriate recipient veins for the RFFF, a viable option or good alternative can be the semi-pedicled RFFF with only arterial anastomosis [12,13]. The cephalic vein can be dissected up to the clavicle, the skin in that region can be tunneled and the RFFF can be pulled through to the head and neck defect [14].

According to the literature, the usual anatomical variant which the ulnar and radial arteries originate from, a common trunk of the bifurcation of the brachial artery, is seen in only 70% of cases [15]. During fetal development, first, the brachial artery branches into the capillary network, and then the definitive arterial pattern of the upper limb is formed as a result of the vascular channel differentiation during capillary remodeling, which can explain arterial variation [16,17].

It was shown that the radial artery can display a high anatomical origin from the brachial artery, or can even branch from the axillary artery [18,19]. The high origin of the radial artery is mostly termed brachioradial artery, but there are several different terminologies: a high bifurcation of the brachial artery; a radial artery originating from the brachial artery; the continuance of the superficial brachial artery as the radial artery; a double brachial artery [20,21,22]. This occurrence of the brachioradial artery is not related to the side of the body or to gender. The prevalence of the high origin of the radial artery, according to different authors, varies from 5% to 15% [23]. The artery mostly originates from the medial circumference of the brachial artery, and less frequently from the axillary artery. The variable anastomotic artery between the brachioradial and “normal” brachial artery, called a cubital crossover, can be found in 18% to 26% of the limbs with a high origin of the radial artery [24].

In our particular case, a preoperative ultrasound and an Allen test showed normal blood flow and normal communication between the ulnar and radial system. Additionally, the ultrasound did not detect the high origin of the radial artery. This could be due to the fact that the protocol of the preoperative ultrasound and its report is to focus on the diameter and flow/obstruction of the postcubital arteries, while the location of the bifurcation previous to this case was not significant. Nevertheless, since the radial artery is often used in vascular, plastic and reconstructive surgery, as well as for arterial puncture and cannulation (transradial access), knowledge of its variations should be of great clinical significance. Furthermore, while this ultrasound oversight could be seen as an error, on the other hand, it improved the ultrasound protocol in our institution and now we regularly screen the anatomical variations of brachial artery bifurcation.

In our experience, during previous RFFF harvests, we did not encounter a situation where the branching of the radial artery was so high in the axillary region. Based on our theoretical knowledge of semi-free flaps, we concluded that it is possible to perform the dissection of the flap pedicle (radial artery, venae comitantes and cephalic vein) till the axillary region in such an unusual situation. The postoperative course was without any complication and the healing of the reconstructed intraoral defect was uneventful.

The main limitation of this report is its limited possibility of generalizing these findings due to only one case. Furthermore, even though our “unorthodox approach” to the high origin of the radial artery resulted in good outcomes, on the other hand, our possible subjective bias and many confounding effects could have influenced the end result.

## 4. Conclusions

In conclusion, RFFF is still of great importance for head and neck reconstruction due to its simplicity in harvesting and its versatility. However, during the preoperative radiological ultrasound, besides the usual characteristics such as the radial artery diameter, flow and possible obstructions, it is also important to explore whether there are any other anatomical abnormalities which could influence the operation. Based on the prevalence of the high origin of the radial artery according to the literature (from 5% to 15%), we have to be aware of that possibility. Hence, a more detailed ultrasound or MSCT angiography is needed to anticipate such situations. Lastly, even though this high origin is uncommon, it also opens a possibility for patients who, due to comorbidities, irradiated neck or low diameter/obstruction of the radial artery, are not eligible for RFFF. Henceforth, a one-act pedicled radial forearm flap is a possibility for them.

## Data Availability

The original contributions presented in this study are included in the article. Further inquiries can be directed to the corresponding authors.
